# Editorial: Xenosensors as the targets of endocrine-disrupting chemicals

**DOI:** 10.3389/fendo.2025.1611152

**Published:** 2025-05-12

**Authors:** Nasrin Ghassemi-Barghi, Fazlullah Khan, Zahra Bayrami

**Affiliations:** ^1^ Pharmaceutical Sciences Research Center (PSRC), Tehran University of Medical Sciences (TUMS), Tehran, Iran; ^2^ Department of Pharmacology, Faculty of Pharmacy, Capital University of Science and Technology, Islamabad, Pakistan

**Keywords:** xenosensors, endocrine-disrupting chemicals (EDCs), environmental pollution, detoxification, biotransformation, signaling pathway, molecular mechanism, gene expression

Endocrine-disrupting chemicals (EDCs) are exogenous substances in the living system known as xenobiotics that imitate the authentic ligands of endocrine receptors and affect the function of the endocrine system, potentially causing health problems. These compounds encompass a wide range of synthetic and natural chemicals, including pesticides, pharmaceuticals, plastic additives, and phytochemicals. Exposure to xenobiotic compounds may elicit several responses at the cellular level, ranging from signaling and adaptation to cell death. These compounds bind to various cellular proteins, which can lead to activation or inhibition of molecular responses that trigger biosynthesis, metabolism, transport, elimination, or binding to various receptors that may cause harmful effects. EDCs can be defined as xenobiotics that can mimic hormones, interfere with endogenous hormone receptors, and disrupt the normal function of the endocrine system. Generally, these compounds are lipophilic and can attach to lipids and accumulate in tissues more than other xenobiotics. A variety of cellular receptors that are responsive to xenobiotics, including EDCs, are termed xenobiotic receptors or xenosensors (1–3).

Xenosensors interact with absorbed xenobiotics and upregulate the transcription of genes encoding xenobiotic-metabolizing enzymes. This enzymatic machinery protects cells and organs by eliminating foreign compounds. The metabolization and elimination occur through different reactions, traditionally classified into three stages. The first stage, Phase I, includes reactions that increase the hydrophilicity of lipophilic xenobiotics like EDCs and prepare them for conjugation reactions in Phase II. Conjugating enzymes transform the metabolites from phase I and unchanged xenobiotics into more polar compounds that can be eliminated in the next stage. Phase III enzymes bind to conjugated metabolites and transport them to bile and urine to be excreted from the body (2). Therefore, xenosensors are considered structures targeted by xenobiotics, including EDCs, that trigger a defensive response of the body at the cellular level. These receptors possess a broad sensitivity to different foreign compounds, including EDCs, and each compound can be detected by several xenosensors (4).

Generally, xenosensors are classified into four classes: Aryl hydrocarbon receptor (AhR), Peroxisome proliferator-activated receptor (PPAR), Constructive androstane receptor (CAR), and Pregnane X receptor (PXR) (5). Further to these receptors, some hormone receptors such as estrogen receptors (ER), estrogen-related receptor (ERR), androgen receptor (AR), and thyroid receptor (TR) have demonstrated activity as xenosensors. Further studies may lead to identifying more nuclear and hormone receptors like the glucocorticoid receptor (GR) and the farnesoid X receptor (FXR), as targets of EDCs (6).

Investigations into gene expression alterations in cells during exposure to EDCs could be advantageous to clarify the role of targeted xenosensors and related pathways as well as identify downstream target proteins that can be applied as biomarkers for detecting body exposure to related EDCs and exploring new EDCs (7).

Alva-Gallegos et al. studied ERα-related mRNA and protein expression in a breast cancer cell line exposed to 22 compounds and showed that 7 small phenolic compounds (3-methylcatechol,4,5-dichlorocatechol, 3,5- dichlorocatechol, 4- fluorocatechol, 4-nitrocatechol, 4- ethylguaiacol, and 4-chlorocatechol) interfere with ER and can be considered potential endocrine disruptors. Another catechol compound, 4-chloropyrocatechol, exhibited partial effects on ER in this study.

Brown et al. performed an integration of various preclinical models (in-silico, *in vitro*, and *in vivo* experiments) to reveal the endocrine-disrupting effect of cannabidiol, one of the main phytochemicals of cannabis. The computational docking method and site-directed mutagenesis assay showed suggestions of interactions between cannabidiol and PXR. *In vitro* experiments defined cannabidiol as a selective agonist of PXR, particularly in humans. According to the *in vivo* experiments, exposure to cannabidiol in mice activated PXR signaling, leading to higher cholesterol uptake by intestinal cells and elevated plasma cholesterol levels.

Pan et al. reviewed the adverse effects of the ubiquitous EDCs on reproductive system health. The underlying mechanisms and pathways of EDCs’ impact on reproductive organs, including receptor-mediated mechanisms, were provided comprehensively. The authors summarized the existing knowledge on the EDCs’ disruptive effects on reproductive disorders and cancers as well as the weakness of evidence for a definitive explanation.

CARs function as potential targets for ECDs and their metabolization has been explored by (De Battistis et al.). In this study, the authors introduced CARs as the main modulator of lipids and their key role in the mechanism of EDCs toxicity and metabolic syndrome, although they have suggested more research is needed to clarify their mechanisms thoroughly. Moreover, they have recommended more studies, particularly systematic reviews, to fill the gaps in the relationship between CARs and the adverse effects of EDCs.
